# Assessment of knowledge, attitude and risk behaviors towards HIV/AIDS and other sexual transmitted infection among preparatory students of Gondar town, north west Ethiopia

**DOI:** 10.1186/1756-0500-4-505

**Published:** 2011-11-21

**Authors:** Yitayal Shiferaw, Agersew Alemu, Amanuel Girma, Afera Getahun, Andarge Kassa, Alemayehu Gashaw, Abebe Alemu, Takele Teklu, Baye Gelaw

**Affiliations:** 1 Department of Medical Laboratory Science, College of Medicine and Health Science, University of Gondar, Gondar, PO Box ET196, Ethiopia; 2 Department of Physiology, College of Medicine and Health Sciences,University of Gondar, Gondar, PO Box ET196, Ethiopia; 3 Department of Biochemistry, College of Medicine and Health Sciences, University of Gondar, Gondar, PO Box ET196, Ethiopia

## Abstract

**Background:**

The first case of HIV in Ethiopia was reported in 1984. Since then, HIV/AIDS has become a major public health concern in the country, leading the Government of Ethiopia to declare a public health emergency in 2002. Although the epidemic is currently stable, HIV/AIDS remains a major development challenge for Ethiopia. The spread of HIV in any community is in part determined by the knowledge of attitude towards sexuality of its members and by their actual sexual practices. The aim of the study was to assess students' knowledge, attitudes and practices regarding HIV/AIDS and STDs in Gondar, North West Ethiopia.

**Methods:**

A cross sectional study was conducted between February 1 to March 1, 2009 in preparatory high school students. Pre-tested questioner was used to generate the data and analysis was made by SPSS version 15. Chi -square value was calculated and p-value < 0.05 was considered statistically significant.

**Results:**

All the students had heard about AIDS before the interview. Knowledge on some aspect of the disease was quite low in the study group. Only half of the students knew that at present, AIDs is incurable and that HIV infection can be acquired through sexual contact with a 'familiar' person. Knowledge about STI was also quite low, 39% knew that pus in the urine is a symptom of STI and 45.4% knew that acquisition of other STIs is increases the chance of HIV transmission following unsafe sex with known cases. 25% of the study group had previous sexual intercourse and exposed at least one risk behavior. About 34% of the respondents had negative attitude towards AIDS and STDs.

**Conclusion:**

Awareness about STDs and methods of prevention of HIV and STDs was low. More risk behavior was observed in male and those with alcohol and drugs of abuse.

## Background

Since the first HIV case was recognized in the United States in 1981, HIV has spread rapidly throughout the world. Statistics from the Joint United Nations Programme on HIV/AIDS (UNAIDS) and the World Health Organization (WHO) in 2006 reported the number of people living with HIV at the end of 2005 were 38.6 million. An estimated 4.1 million were newly infected with HIV and 2.8 million lost their lives due to AIDS. Although the latest UNAIDS and WHO estimates were lower than those published in the AIDS epidemic update-December 2005, the number of people living with HIV continued to rise [1].

The first case of HIV in Ethiopia was reported in 1984 [2]. Since then HIV/AIDS become a major public health concern in the country leading the Government of Ethiopia to declare a public health emergency in 2002. In 2007, the estimated adult HIV/AIDS prevalence in Ethiopia was 2.1 percent. Again National projections estimate approximately 1.1 million Ethiopians are living with HIV and prevalence will increase slightly to 2.3 percent by 2009 [2]. Although the epidemic is currently stable, HIV/AIDS remains a major development challenge for Ethiopians. Poverty, food shortages and other socio-economic factors amplify the impact of the epidemic [2].

Over half of all new infections worldwide were among young people between the ages of 15 and 24. Every day, 6,000 young people become infected with HIV- more than five every minute [3].

In the United States alone, half of all new infections are estimated to be among people under age 25 years and the majority of young people are infected sexually [4]. Demographic health surveys of many countries have prevailed that adolescents in now a days are experienced puberty at younger age than the previous generation. As result, they are involved in early initiation of sexual intercourse; most of it is being unsafe, unplanned and exposing them to unwanted pregnancy, abortion and sexually transmitted disease [5].

Risk behaviors like unprotected sex, multi partnership, no or inconsistence use of condoms and drug of abuse are extremely determinate to health of adolescents and young adults putting them at high risk to HIV/AIDS and other Sexual transmitted diseases (STDs) [5].

Girl adolescents are particularly vulnerable to HIV/AIDS and other STDs. Studies indicated for every 15-19 years old boys there are 5-6 girls of the same age infected with HIV. This is often exacerbated by the fact that most young women are likely to be having sex with men older than themselves due to economical like "Sugar daddy" relation ship in many African cities. Another serious issue is younger girls lack sexual negotiation because of fear of physical abuse, rejection and their partner objection [6].

Other important factors for the spread of HIV in Africa today are alcohol and drugs of abuse. The influence of alcohol and experimentation with drug promotes increase in the incidence of high-risk behaviors in particular sex. Sexual offense such as rape usually committed under the influence of alcohol or drug [7].

The spread of HIV in any community is in part determined by the knowledge of attitude towards sexuality of its members and by their actual sexual practices. Before formulating public health policies for the prevention of HIV, it is critical to obtain information about the prevalent knowledge, attitude and practice (KAP) regarding HIV/AIDS, other STDs and sexuality in the target community. Several studies on KAP regarding HIV/AIDS have been reported from different parts of Ethiopia. However; none of them addressed the increased risk of HIV infection associated with acquisition of other STDs. In addition studies on KAP regarding HIV/AIDS and other STDs in Gondar, North West Ethiopia are insufficient and there must be a continuous assessment of KAP regarding HIV/AIDS and other STDs in a community to observe the impact of different awareness creation forums. Therefore; the aim of the study was to assess students' knowledge, attitudes and practices regarding HIV/AIDS and other STDs. We also investigated the impact of gender and family income of the students on their knowledge, attitude and practice.

## Methods

### Study area and Design

A cross sectional study was conducted between February 1 to March 1, 2009 in Fasiledess preparatory school, which is found in Gondar town with estimated residents of 250,000 populations. Gondar town is located 730 km away to the North West from the capital city, Addis Ababa. The school prepares students for National collage entrance examination.

### Study population and sampling procedure

There were a total of 658 preparatory students (grade 11 and grade 12) in Fasiledess preparatory school during the study period distributed into 11 sections (6 sections (A-F) for grade 11 and 5 sections (A-E) for grade 12). The number of students between sections were varies ranging between 50 and 70. Assuming homogeneity in academic status among students in the same grade, a total of 240 students were recruited for the study. Stratified proportionate sampling was used to select study participants. Shortly, students' list was obtained from student registration books and stratified into grades. Five sections were selected randomly (3 sections from grade 11 and 2 from grade 12) from stratum and of which proportionate numbers of students were selected using systematic random sampling to obtain the total sample for the study.

### Data collection

The study instrument was a self-administered questionnaire which comprised of four parts. Part- A related to student's sociodemographic background, Part- B on knowledge regarding HIV and other Sexual transmitted infections (STIs), Part- C on students' attitude scale towards HIV/AIDS and Part -D on high risk behavior or practice related to HIV& other STIs transmission.

The knowledge, attitude and practice questionnaire was modified from the instrument used by a survey on HIV/AIDS knowledge, attitude & practice (KAP) reported by the Department of Education, Free State South Africa (2006) which was adopted from the WHO AIDS Questionnaire (WHO 1990).

Knowledge was assessed using a 31-item questionnaire which includes knowledge on STDs, ways of risk reduction for HIV transmission, predisposing risk behavior and practice to HIV and other STIs, symptoms of STDs other than HIV/AIDS, treatment of AIDS, prevention methods of HIV and other STIs and the contribution of other STDs acquisition for the spread of HIV infection.

Attitude was assessed using a 10-item questionnaire which includes attitude: towards screening of HIV and other STIs, towards AIDS patients, towards gender equality as related to sexuality and towards visiting clinics for cases of STDs other than HIV/AIDS. The questions on high risk behaviors had 11 items related to unprotected sex and needle sharing.

The English questionnaire was translated into simple Amharic (local language) and back translated into English. Pre- test of questionnaire was done on twenty students and the result was used to improve the phrasing of questions in the questionnaire. Questionnaire validation tests showed that the Alpha Cronbach was 0.87 for knowledge, 0.71 for attitude and 0.72 for risk behaviors.

### Scoring

For knowledge, each right response was given a score of 1 while a wrong or unsure response was scored 0. Total knowledge scores can range between 0-31. Knowledge scores from 0 to 15 were considered as poor knowledge while knowledge scores more than 15 was considered as having good knowledge regarding HIV/AIDS. Attitude towards HIV/AIDS patients was assessed using a 10-item questionnaire where attitude scores between 0 to 5 were considered as negative attitude, and scores 6 to 10 were considered as positive attitude. High risk behavior or practice was assessed using an 11-item questionnaire where report of at least one negative behavior related to HIV transmission is considered as having high risk behavior.

### Data management and analysis

During data collection process, the data were checked for completeness and any incomplete or misfiled questions were sent back for correction. Data were double entered and analyzed using SPSS-15 statistical software (SPSS Inc. Chicago, 2007). Descriptive statistics were used to give a clear picture of background variables like age, sex and other variables in well structured questionnaire. The frequency distribution of both dependent and independent variables were worked out. The association between variables was measured and tested using chi square and odds ratio. P-value < 0.05 was considered as significant in all cases.

### Ethical consideration

Ethical clearance was obtained from University of Gondar collage of Medicine and Health Science Ethical Review Board. Prior to data collection, all study participants were given information on the study and assured that all data is confidential and will only be analyzed as aggregates. All respondents signed the informed consent form before participation.

## Results

### Knowledge about HIV/AIDS and STDs

Two hundred forty students agreed to participate and completed the questionnaire after open recruitment of students in the selected sections at the school. The mean age of the 240 respondents was 19.5 ± 5.0 years and ranged from 15 to 24 years. 125 (52.1%) were male and result male to female ratio of 1.09:1. All of the students were single and 141(58.7%) were living with both parents, 47(19.6%) with single parent, 45(18.8%) alone in rented house and 7(2.9%) were living with relatives. With regards to socioeconomic, 131(54.6%) were obtained from low and 109 (45.4%) from high socioeconomic class families.

All the students had heard about AIDS before the interview. The source of information were radio (50%), television (46.7%), News paper (33.3%), teachers (25%), parents (21.7%), health workers (13.3%) and youth club (11.7%) where more than one sources were common.

Generally students were well informed about select aspects of HIV/AIDS (Table 1). Thus, a very high (> 80%) proportion of students were conversant with the major modes of spread of HIV and with utility of condoms in preventing HIV infection. Nonetheless, only half of the students knew that at present, AIDs is incurable, and that HIV infection can be acquired through sexual contact with a 'familiar' person. There was little misconception like HIV spread by bite of mosquitoes and sharing of equipments. While both sexes were equally misinformed about the risk of getting HIV by mosquitoes and sharing of equipments, more girls' belived that HIV/AIDS was curable and that sexual contact with a 'familiar' person was risk-free.

**Table 1:** Knowledge about HIV/AIDS, prevention of HIV and other Sexual transmitted infections

		Correct responses(%) according to				
		Gender		Family income		
		
Statements	Correct response	Male(125)	Female(115)	Low(131)	high(109)	Total (240)
**Knowledge about HIV/AIDS**						
HIV Spread through sexual intercourse	False	96	98.1	97.7	96.7	97.1
HIV Spread from pregnant mother to child	True	60	53.9	56	58.8	57.1
AIDS is a curable disease, if diagnosed in early stage	False	52	47.8	53.4	47.1	50
HIV spread from sharing of bath room, clothes and kissing	False	97.5	97.6	98.7	97.6	98.3
HIV spread through mosquito bites	False	99	97.7	98.5	98	98.3
**Knowledge about predisposing factors for HIV infection**						
HIV not prevent by sex with a person who you know very well	True	52	47.8	47.1	53.4	50
Condom prevent HIV	True	75.6	75	80	70.4	75.4
Avoid sex with commercial sex workers	True	31.5	29.5	28.5	32	30.4
Avoid alcohol and drugs of abuse	True	3.5	3	2.5	3.5	3
Sexual abstinence	True	78	82.6	79	81	80
**Knowledge about other STIs**						
Condom prevent STIs	True	74.4	76.5	75	77	76
Other STDs +risk sexual behaviors increased chance of HIV acquisition following contacts with known cases	True	48	44	50	42	46
Ulcers and/or pus in the genital organ and pain on passing urine are some of the features of STDs other than HIV/AIDS	True	55	21	38	39.5	39

Mean (SD) knowledge score

19.97 (6.37)

Max = 31, min = 6

All knew that AIDS is STD. However; a striking finding of this study was the poor knowledge of the students regarding symptoms of common STDs other than HIV/AIDS, the increased risk of HIV infection associated with acquisition of other STDs and the impact of drugs of abuse and alcohol for the spread of HIV and other STIs.

### Attitudes towards HIV/AIDS and STDs

About 34% of the respondents had negative attitude towards HIV, AIDS patients and other STDs. More than 30% of the students associated AIDS with an immoral life style and even recommended isolation of AIDS patients. Half of the students favored for screening of HIV and STIs. However; third of the students was not welling to visit infection control clinic following acquisition STDs other than HIV/AIDS. More girls than boys belived that banning a commercial sex could control the spread of HIV and other STIs (Table 2).

**Table 2:** Attitudes towards HIV/AIDS/other STDs and gender equality as related sexuality

		Responses indicating desirable attitude(%) according to				
		
		Gender		Family income		
Attitudes	Appropriate response	Male(125)	Female(115)	Low(131)	high(109)	Total (240)
**Towards HIV/AIDS/STDs**						
Only those people who lead immoral lives will get HIV	Disagree	63	68	67	64	65.4
AIDS patients pay the price for their immoral life	Disagree	42	38	38	42	40.4
AIDS patients should be isolated for the safety of others	Disagree	69	71	74	66	70
Banning prostitution can control the spread of HIV& other STDs	Disagree	18	50	49	19	33.8
HIV/AIDS is sever and more affects youth	Agree	72	93.9	84	80	82.5
Go to clinic for STDs	Agree	30	35	40	24	32.5
Screening for HIV and other STDs is good	Agree	45	55	55	45	50
**Towards gender equality as related to sexuality**						
Women are more responsible than men for prostitution	Disagree	48.4	51.6	52.5	47	50
A man can have premarital sex, but a women should not	Disagree	81	80.5	79	82.5	80.8
It is better for men to have sexual experience before marriage	Disagree	79.6	83.7	81.8	79.5	81

Mean (SD) Attitude score

7.1 (2.25)

Min. scores 2- max. score 10

### Bivariate analysis

In Bivariate analysis, significant more negative attitude (responded less than half of the favorable attitude questions) were observed in male (OR = 2.6; p > 0.001) and students obtained from high socioeconomical class (OR = 1.2; p > 0.005). However; being knowledgeable (responded more than half of the knowledge questions) did not significantly associated with gender and socioeconomical status of the family (Table 3).

**Table 3:** Association between knowledge and attitude with gender and socioeconomical characteristics

		Attitude				
					
Sociodemographic characteristics	N	Positive	Negative	OR	Χ^2^	p-value
Gender						
Male	125	69(55.2%)	56(44.8%)	2.6	1.52, 4.62	0.001
Female	115	89(77.4%)	26(22.6%)			
Socioeconomic class						
Low	131	114(87%)	17(13%)	1.2	1.05, 1.37	0.005
High	109	79(72.5%)	30(26.5%)			
Sociodemographic characteristics		Knowledge status				
		Good knowledge	Poor knowledgeable			
Gender						
Male	125	106(84.8%)	19(15.2%)	1	0.51, 2.11	0.928
Female	115	98(85.2%)	17(14.8%)			
Socioeconomic class						
Low	131	114(87%)	17(13%)	0.8	0.37,1.55	0.440
High	109	91(83.5%)	18(16.5%)			

### Risk behaviors

Table 4 shows that 25% of all respondents had at least one risk behavior related to HIV & other STIs transmission. 65% of male and 35% of female already initiated sexual intercourse at mean age of 17.3 and17.1 years respectively (Result not presented here). About 39% reported having unprotected sex (sex without condom); 43.3% of sexually active students had more than one sexual partner; 5% reported having sex with sex workers; 15% reported having sex under the influence of alcohol; 31.7% undergo sexual intercourse in unusual route and 5% shared sharps.

**Table 4:** Risk behavior and practice that predispose students towards HIV and other STIs

Risk behavior and practice	Frequency (%)
At least one risk behavior & practice	
Yes	60(25)
No	180(75)
Shared sharps	
Yes	12(5)
No	228(95)
Sexual initiation	
Yes	60(25)
No	180(95)
Sexual partner during sexual initiation	
Fixed friend	19(31.7)
Causal friend	38(63.3)
CSW	3(5)
Number of sexual partner	
Single	34(56.7)
Multiple	26(43.3)
Condom use during sexual intercourse	
Always	37(61)
Sometimes	23(39)
Sex under external influence	
Alcohol	9(15)
drugs	8(13.3)
Route of sexual intercourse	
Usual anatomical sites	41(68.3)
Un usual anatomical sites	19(31.7)

Further analysis indicated significantly more risk behaviors that can predispose students to HIV and other STIs were practiced in male, alcohol users and drugs abusers (p < 0.05). However; practicing risk behavior among students did not significantly varies with respect to their knowledge, attitude and socioeconomical status (Table 5).

**Table 5:** Association between risk behaviors and knowledge, attitudes, gender and socioeconomic status

		At least one risk behavior				
					
Sociodemographic characteristics	N	Yes 60(25%)	No 180(75%)	OR	Χ^2^	p-value
**Gender**						
Male	125	39(31.2%)	86(68.8%)	1.8	(1.15,2.95)	0.009
Female	115	21(18.3%)	94(81.7%)			
**Socioeconomic class**						
Low	131	39(29.8)	92(70.2%)	0.56	(0.31,1.03)	0.061
High	109	21(19.3)	88(80.7%)			
**Alcohol**						
Yes	64	37(57.8%)	27(42.2%)	9.1	(4.7,11.67)	0.000
No	176	23(13.1%)	153(86.9%)			
**Drugs of abuse**						
Yes	26	15(57.7%)	11(42.3%)	5.11	(2.2,11.9)	0.000
No	214	45(21%)	169(79%)			
**Attitude**						
positive	193	47(24.4%)	146(75.6%)	0.842	(0.41,1.72)	0.639
Negative	47	13(27.7%)	34(72.3%)			
**Knowledge status**						
Good Knowledge	205	51(24.9%)	154(75.1%)	0.957	(0.421,2.172)	0.916
poor knowledge	35	9(25.7%)	26(74.3%)			

## Discussion

A very important finding of our study was that all students participated in the survey had heard about AIDS. This finding was in line with the behavioral surveillance conducted in 2002 by Ethiopian Ministry of health (EMOH). Similar comparable result also reported in studies done elsewhere in the country [5,8,9]. This is an encouraging finding which should further be strengthened by establishing additional HIV/AIDS youth clubs in the study area. As reported previously [10], the print and electronic media constituted the most important modes of dissemination of information about HIV/AIDS. Particularly, only 25% of students in our study obtained information about HIV/AIDS from their teachers. Our finding emphasizes the need to improve the role of teachers in HIV/AIDS awareness programmes. This is important because educating school children about safe sex is one of the most ways of postponing the onset of sexual activity among them [11]. Intervention programmes providing sex education in school have been reported to result in a marked improvement in the knowledge of students about HIV/AIDS and have been associated with a positive change in their attitude towards the disease [12].

A further examination on the frequency distribution revealed substantial deficiencies in knowledge of HIV/AIDS in certain key areas. The importance of sexual abstinence in the prevention of HIV infection was unrecognized by18.8% of the students. Half of the participants regarded sex with 'someone they know' as being free of risk from HIV infection and considered AIDS as a curable disease. There were also misconceptions in this study where kissing and sharing equipments with AIDS patients (1.7%) and mosquito bite (1.3%) would transmit HIV. Students in other parts of Ethiopia also reported similar misconceptions [9,10].

While it is encouraging to note that most of the students knew AIDS is STD and was aware of the common modes of HIV transmission, it is disturbing that knowledge of symptoms and the methods of preventing STDs other than HIV were poor. Most students were unaware that individuals having STDs other than HIV and undergo risk sexual behavior have a higher chance of acquiring HIV following contact with known cases compared to individuals without STDs. This discrepancy between knowledge of STDs other than HIV and its impact in HIV spread is not surprising; similar findings have been reported from developed countries [13].

The association between HIV and other STDs needs to be highlighted in educational programmes on HIV/AIDS. This is especially important in Ethiopia because the prevalence of non-HIV STDs has been reported to be very high and it is widely accepted that eradication of STDs at the population level is difficult. An understanding of the link between other STDs and HIV will probably promote safe sexual practices. The overall knowledge status of the study participants was not statistically influenced by gender and socioeconomic level. In contrast to our finding study in Kerala, India indicated good knowledge was observed in male compared to female [14]. This discrepancy may be due cultural milieu where Kerala's women are subject to more social restrictions than men of the same age [14] which was not observed in our study area and perhaps, this contributes to lower knowledge scores in female in their study compared to us.

### Attitudes towards HIV/AIDS

Majority of the respondents (82.5%) perceive the severity of HIV and belived AIDS affects youths at a high proportion than any other group. This is interesting and it should be encouraged as perceived severity and perceived susceptibility are important components for behavioral change. We noted that students' attitudes towards AIDS were combined with their concepts of sexual morality. A majority of the students' belived that AIDS was a consequence of deviation from the 'moral' life. Majority of the students were sympathetic towards AIDS patients and were against isolating AIDS patients from society.

Half of the students' were belived that women was more responsible for prostitution compared to men. Many studies have reported good knowledge on HIV/AIDS but negative attitude is still prevalent [14]. From our study, although students had good knowledge regarding HIV/AIDS, they still harbor negative attitude towards HIV and HIV/AIDS patients. Knowledge alone is not enough to change attitude**s **towards peopl**e **having HIV**/**AIDS, but deep seated social and cultural factors such as religion, attitude towards ill-health and risk behaviors especially sexual behaviors can affect attitude too.

### Risk behavior

In this study, 25% of our respondents reported having at least one risk behavior which predisposes them for HIV and other STIs. This is comparable to study done in Awasa, Ethiopia [10]. The mean age of first sexual contract for females and males were 17.1 and 17.3 years (data not presented) is comparable to the observations reported in previous studies [15,16]. The findings that over 40% of the sexually active respondents had multiple sexual partners including commercial sex workers (CSWs) indicate such risky behavior can predispose the students to acquisition of STDs including HIV. Furthermore; condoms were used only some times by about a third of those who were undergo sexual intercourse. In practice, sexually active students are still shy to buy condom because of religious and socio-cultural norms related to youths, especially unmarried youths. It is worth noting that about 30% of sexually active students undergo sexual intercourse under the influence of alcohol and drugs of abuse and 31.7% undergoes through an unusual route. In-line with this, risky sexual behaviors among college students in association with their condom utilization has recently been reported from Gondar [17]. Summarization of data indicate at least one risk behavior observed more in male, alcohol consumers' and drugs abusers' compared to their counterparts. Similar findings reported in other studies [18].

This study suggests that the education system needs to implement specific and focused educational programs for students in school prior to collage admission and promote health promotion. It is important that school students understand HIV prevention and transmission, as well as develop positive attitude and good practice. The school is a good place and time to have peer education programmes that address self esteem, healthy sexual attitudes, as well as to be socially active, accepting and caring. Taking into consideration the fact that not all students are sexually active, developing messages geared towards them while offering strategies that help students delay sex, refuse sex, or negotiate safer sexual practices should be included. This program must give students an understanding of why it is more advantageous to abstain from sex without promoting unnecessary fear.

## Conclusion

In our cross sectional survey of preparatory students in Gondar, awareness of HIV and its mode of transmission were high. In comparison, awareness about STIs other than HIV and methods of prevention of HIV and STIs were low. There were gender and income differences in attitudes related to HIV/AIDs and sexuality, with female and low income demonstrating a positive attitude. More risk behavior was observed in male and those with alcohol and drugs of abuse.

Therefore, our investigation call for continued and strengthened health education to bring change in knowledge in regard to relationship between HIV and other STDs, promote positive attitude more in boys and brings behavioral changes among the students in Gondar town.

## Conflict of interest

The authors declare that they have no competing interests.

## Authors' contributions

YS, AA, AGa, AGe, AGi and AK conceived the study, undertook data collection, undertook statistical analysis and drafted the manuscript. AA, TT and BG initiated the study and made major contributions to the study design and statistical analysis. All authors contributed to the writing of the manuscript and approved the submitted version of the manuscript.
